# δ^34^S and Geochemical Analyses for the Determination of, and Discrimination between, Salt Samples of Different Geographic Origin: A Feasibility Study

**DOI:** 10.3390/foods12081572

**Published:** 2023-04-07

**Authors:** Micha Horacek

**Affiliations:** 1 AIT-Austrian Institute of Technology GmbH, Konrad-Lorenz-Straße 24, 3430 Tulln, Austria; untertrias@gmail.com; 2 Department of Lithospheric Research, Vienna University, Josef Holaubek-Platz 2, 1090 Vienna, Austria

**Keywords:** food, provenance, sulfur isotope, trace elements, sea salt, rock salt, fleur de sel

## Abstract

The geographic origin of salt is usually regarded as unimportant, as it is a one-quality product produced in vast quantities. However, certain salt brands, especially sea salt (fleur de sel), are sold at significantly higher prices. Thus, it is necessary to control the declared geographic origin of salt. Such controls are already frequently carried out for foodstuffs, but salt is an inorganic commodity. Thus, δ^34^S analysis combined with element concentration analysis was carried out. The results show very similar δ^34^S values for all sea salt samples, which is to be expected due to the homogenous marine δ^34^S value. Still, slightly higher values have been found in Mediterranean salt samples. Rock salt samples show differing δ^34^S values depending on the time they were formed, and if the salt samples are of marine or terrestrial origin. Terrestrial/continental salt samples are characterized by elemental patterns significantly differing from marine ones. However, within marine samples (sea salt and rock salt) there also exist differences enabling the differentiation of samples.

## 1. Introduction

The geographic origin of food has gained more attention in recent years, as consumers are increasingly willing to pay a higher price for regional and national products. Thus, by incorrect declaration of the geographic origin a higher profit can be gained, and control of the declared geographic origin is necessary to prevent consumer deception. As has been shown, the control of accompanying paperwork is insufficient to prevent fraud, control of the food commodity itself is required. The method of choice for this task is usually stable isotope analysis (IRMS: Isotope Ratio Mass Spectrometry, e.g., [1,2,3,4,5,6,7], among many others). However, stable isotope analysis investigates the main elements of biogenic material (H, C, N, O, S). Thus, as salt is an abiotic material consisting almost exclusively of NaCl for stable isotope analysis (IRMS), only the traces of sulfur in the salt can be measured for its isotope ratio.

Generally, salt is regarded as a “non-brand” one-quality product by most of the consumers. Salt is produced industrially and in huge quantities without differences in quality; thus, its geographic origin was of no interest. However, several new salt products have been introduced to the market. In addition to mixtures of salt with various spices, different kinds of salt with the indicated geographic origin are now also available at significantly higher prices than “ordinary” (refined) salt. Often it is sea salt with crystals forming at the water surface, which is called “fleur de sel” (salt flowers). In this work, we want to investigate how we can differentiate the different kinds of salt, for which differentiation is possible, and the reasons for “individualism” of the different origins.

There are two “groups” of salt: rock salt (ancient sea salt) and sea salt (newly formed/modern marine salt). Another group consisting of non-marine newly formed salt is also identified whereby any landlocked location without direct connection to the open sea falls into this group, e.g., Dead Sea salt. A fourth group of ancient non-marine salt also exists and in this investigation it is assumed that Kalahari salt (South Africa) falls into this category.

**Figure 1:**
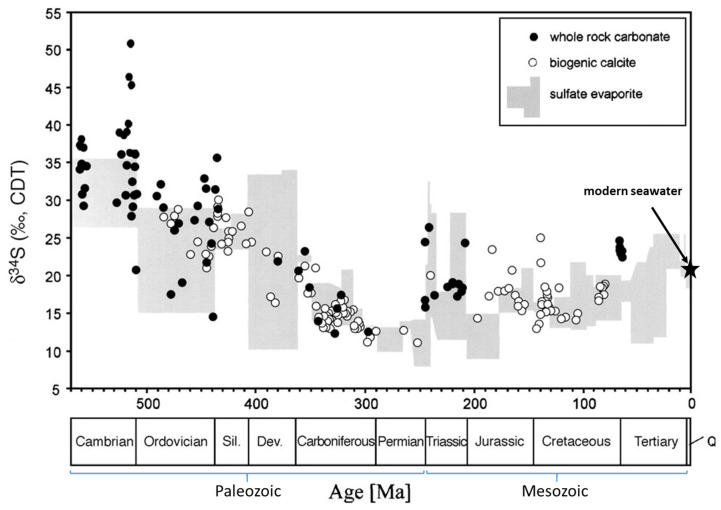
Marine δ^34^S evolution versus geological time scale (after [11]). Asterisk marks modern sea water δ^34^S value. Q: Quarternary.

The geological marine ^34^S curve (Figure 1) has been investigated and accumulated by Holser 1977 [8]; Claypool et al., 1980 [9]; Cortecci et al., 1981 [10]; and Kampschulte and Strauss 2004 [11], among others. In Austria, Spötl and Pak 1996 [12], investigated the Alpine salt deposits’ ^34^S values and demonstrated a significant difference between the Permian and Triassic deposits. Horacek et al., 2010 [13], showed the rapid change from the low uppermost Permian (and lowermost Triassic) to the very elevated marine ^34^S values already in the Lower Triassic. Therefore, it is assumed that δ^34^S is an important proxy for the differentiation of salts of different type and origin.

This study is dealing with unrefined, raw salt, as the high-priced salt products are marketed and advertised to possess certain special properties due to their “pure/pristine” and unrefined status. For comparison, one refined salt sample was included (Toskana rock salt). A small increase in Mg and/or K in this sample might result from the addition of iodate and improvers of pourability.

Previously, Dufosse et al., 2013 [14], documented the successful discrimination between sea salt of different salt gardens at the French Atlantic coast by bacterial communities. Sharma et al., 2021 [15], investigated the microbial community of saffron corms to differentiate the different geographic origins of these corms; thus, it is interesting to see that the bacterial community of abiotic commodities can be used for the differentiation of geographic origin. Tchaikovsky et al., 2019 [16], and Epova et al., 2019 [17], noted differences in salt ^87^Sr/^86^Sr ratios; however, neither work gives a detailed description of all locations of the origin of the salt, as other commodities (caviar and ham) were the foci of these studies. Still, the ^87^Sr/^86^Sr ratio of salt is another interesting and potentially relevant proxy to differentiate food and salt origin (also see Horacek et al., 2022 [18], and Horacek, 2022 [19]); however, it was not applied in the present study.

In this study, I hypothesize that a differentiation of the investigated salt samples by their element concentrations and δ^34^S ratio should be possible.

## 2. Materials and Methods

A total number of 14 salt samples were investigated. Twelve salt samples from throughout the world (Italy, Portugal, Spain, Pakistan, Korea, New Zealand, Mauritius, South Africa, Slovenia, Israel) were collected. Out of these salt samples 8 were sea salt samples in the sense of coming from water bodies that are a part of the open seas and oceans (Italy, Portugal, Spain, Korea, New Zealand, Mauritius, Slovenia) and 4 samples were rock salt (Italy, Pakistan) or had precipitated from non-marine water (South Africa, the Dead Sea). Additionally, 2 salt rock samples from 2 Austrian salt mines (Hallein (HA) and Altaussee (AA)) were analyzed (Table 1). From each of these two latter salt rock samples, two subsamples were taken and analyzed. The subsamples were further investigated by using different kinds of mills for grinding and filtering the samples after dissolution in water before re-precipitation. The latter sample subset is not of specific interest to the present investigation and was only included for the sake of completeness.

### 2.1. Sulfur Isotope Analysis

For sulfur isotope analysis, salt samples were dissolved and sulfate precipitated as barium sulfate by adding 1 molar barium-chloride solution (for a detailed description see Horacek et al., 2010 [13]). Precipitate was collected in a filter and washed with deionized H_2_O. The barium sulfate precipitates were weighed into tin capsules and introduced into an elemental analyzer (Vario, Elementar, Hanau) that was connected via a Con Flo (Thermo, Bremen) with an isotope ratio mass spectrometer (Delta xp, Thermo, Bremen). Results are reported in the conventional δ notation as deviations in ‰, with respect to the internationally accepted V-CDT (Vienna Canyon Diablo Troilite) standard. Reproducibility was better than ±0.4‰ (1 σ).

### 2.2. Trace Element Analysis

Analyses for the concentrations of trace elements were performed by applying inductively coupled plasma mass spectrometry (ICP-MS) using a Perkin Elmer Sciex Elan 6100 instrument. Aliquots of each salt sample were diluted with high purity nitric acid. For calibration, ICP multi-element standard solution VI for ICP-MS (30 elements in dilute nitric acid), Certipur^®^, and Spex CertiPrep CLMS-1 Claritas PPT^®^ Grade ICP-MS Multi-Element Solution for rare earth elements were used. Rhodium was used as an internal standard.

## 3. Results

### 3.1. δ^34^S

**Figure 2:**
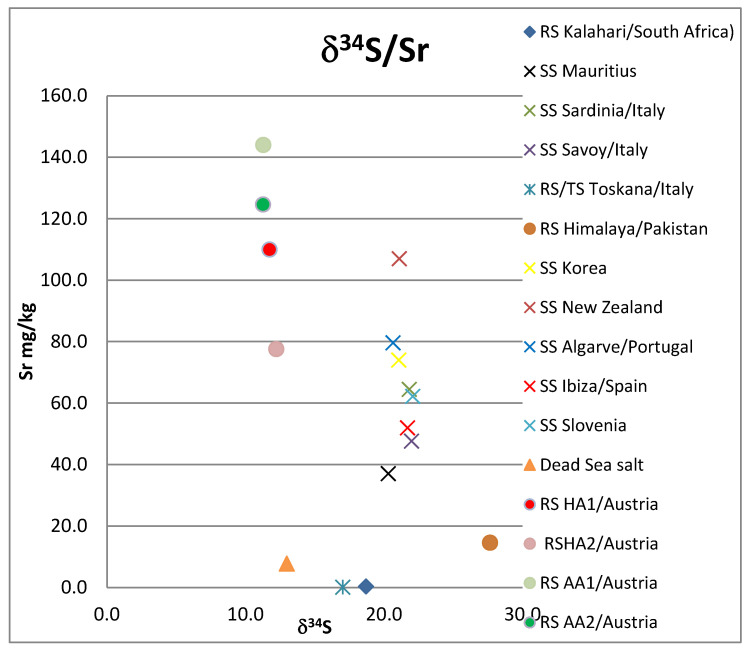
δ^34^S versus Sr concentration. RS: rock salt, SS: sea salt, TS: table salt, HA: Hallein, AA: Altaussee.

The sea salt samples all range between 20.3 and 22.1‰ V-CDT (Figure 2). The salt samples from the Mediterranean Sea show slightly elevated values between 21.7 and 22.1‰. The sea salt samples from the rest of the world range between 20.3 and 21.1‰. The rock salt samples range from 11.3 to 27.7‰, the Dead Sea sample has a δ^34^S signal of 13.0‰, and the Kalahari salt possesses a δ^34^S value of 18.7‰ (see Figure 2 and Table S1 (Supplementary Materials).

### 3.2. Element Concentrations

**Figure 3:**
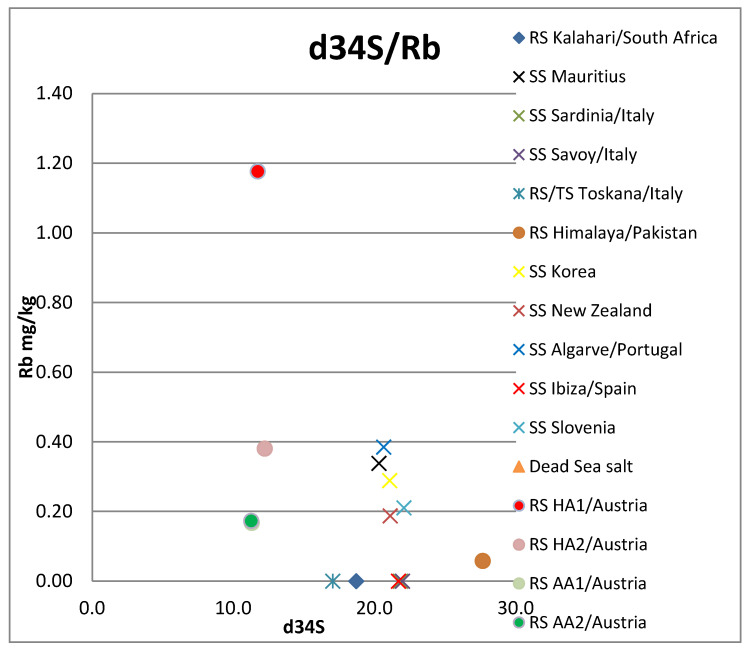
δ^34^S versus Rb concentration. Note that the symbol of the Dead Sea sample is outside of the diagram, as the sample contains 139 mg/kg Rb. RS: rock salt, SS: sea salt, TS: table salt, HA: Hallein, AA: Altaussee.

**Figure 4:**
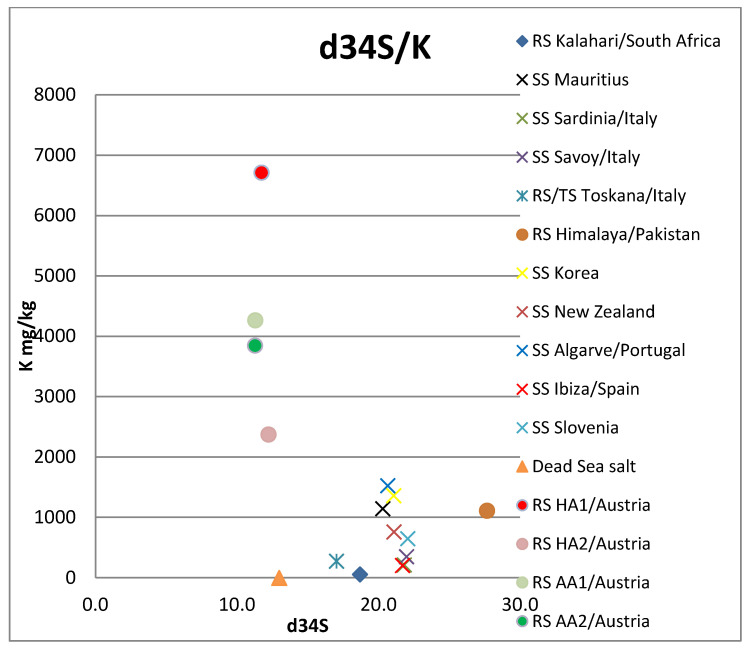
δ^34^S versus K concentration. Note that the symbol of the Dead Sea sample is outside of the diagram, as it contains 154,667 mg/kg K. RS: rock salt, SS: sea salt, TS: table salt, HA: Hallein, AA: Altaussee.

**Figure 5:**
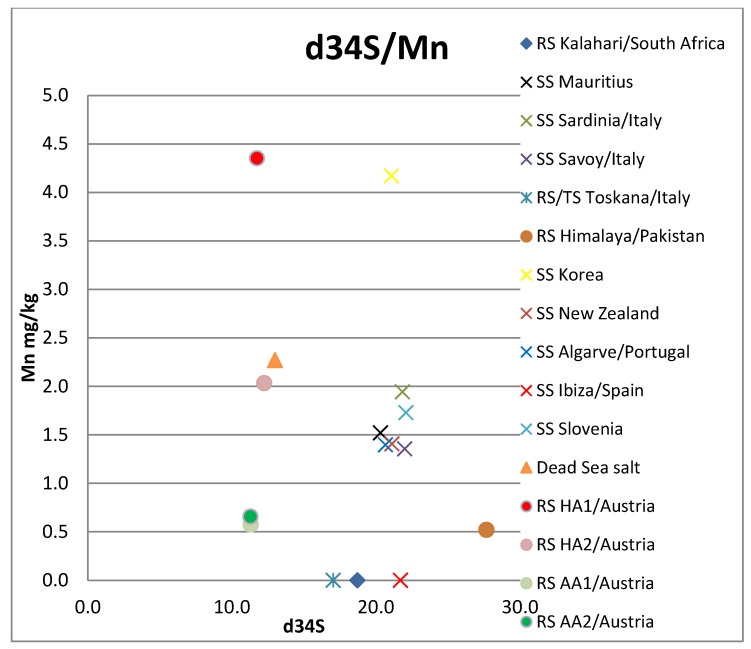
δ^34^S versus Mn concentration. RS: rock salt, SS: sea salt, TS: table salt, HA: Hallein, AA: Altaussee.

Of the elements analyzed, the following were below the detection limit for all samples: Be, Cr, Fe, Zn, Ga, Nb, Ag, Sn, Te, Pr, Nd, Eu, Gd, Tb Dy, Ho, Er, Tm, Yb, Lu, W, Bi. Elements As and Se were only detected in one sample (Dead Sea salt), also: Fe, Tl, Cu, Sb, Gd, Sm (Hallein salt sample); and Cd (Korean salt sample). All other investigated elements occurred in at least two samples (see Table S1). The Dead Sea/Israel salt sample is strongly enriched with the elements Rb, K, and Mg. Y only occurs in the two Austrian rock samples (Figure 2, Figure 3, Figure 4 and Figure 5, Table S1).

Weak positive correlations in element concentrations are present for Ca and Mg (R^2^ ~ ca. 0.6) and Mg and K (R^2^ ~ 0.75) for all of the salt samples except for the Austrian rock salt samples.

Due to milling in a corund mill of the subsamples of the Austrian rock salt samples, an increase in Al is noted (but no other increase), easily explained by abrasion from the mill. Potentially Toxic Element (PTE) concentrations in salt from two lakes in Iran have been analyzed and studied by Mostafaii et al., 2022 [20], and report similar ranges of magnitude for the elements As, Cd, Ni, and Pb. The authors explain that monitoring of the PTEs is important to protect consumers from health risks. With respect to the investigated salt samples, the Dead Sea salt sample contains a notable As concentration (7.74 mg/kg).

## 4. Discussion

### 4.1. δ^34^S-Results

Generally, large differences in δ^34^S exist in rocks (e.g., see Horacek and Cannavan, 2022 [21], and the references therein). However, the marine d^34^S value only varies within a rather narrow range between + 10 and + 40‰ within the Phanerozoic [9,11]. Thus, the δ^34^S results of the salt samples mirror the marine sulfur isotope value at the time of salt precipitation, except for the two non-marine salts (South Africa and the Dead Sea/Israel). In the latter two cases, the ^34^S values represent the isotopic composition of the sulfate dissolved in these respective water bodies, which in turn are influenced by the geology of the catchment area, from where the sulfate is washed out by the water (and in the present two cases they lie within the marine range). The rock salt samples evidence that samples of different ages possess differing δ^34^S values, as the marine ^34^S value shifted with time and thus the rock salt samples show the respective marine ^34^S value during the time of their precipitation/formation. The rock salt from Pakistan most likely formed during the Paleozoic (Figure 1), where such elevated ^34^S values were common [9,11]. The low values of the Austrian rock salt samples evidence an upper Permian [12] age (well in agreement with the geology), as these low values are almost unique in Earth’s history. The value of the Toskana salt sample hints at a Triassic or Cretaceous age (Figure 1) [9].

The sea salt samples show an interesting feature, as the samples precipitated from the Mediterranean Sea are slightly elevated (and lie around 22‰), with respect to the sea salt samples of other origins (around 21‰). It seems that there might exist a small difference in δ^34^S between the Mediterranean Sea and the other seas (these other samples come from the Atlantic Sea (Portugal), the Indian Ocean (Mauritius) and the Pacific Ocean (Korea and New Zealand)). Theoretically, it is assumed that the marine δ^34^S value is homogeneous throughout the different seas, as sulfur has a very long residence time (ca. 9myr, [22]).

### 4.2. Element Concentrations

In the *Encyclopedia Britannica* [23] it is stated that “…irrespective of the source of the seawater, salt obtained by the evaporation of seawater has the following composition: sodium chloride 77.76%, magnesium chloride 10.88%, magnesium sulfate 4.74%, calcium sulfate 3.60%, potassium chloride 2.46%, magnesium bromide 0.22%, and calcium carbonate 0.34%…”. However, as shown below and in Table S1, the element concentrations vary to some extent, even among the sea salt samples.

The salt from South Africa (Kalahari salt) can be unambiguously identified by the elevated uranium content. Furthermore, the mentioned salt sample also possesses the lowest K, Mg, and Ca values. The K concentration in the Dead Sea salt sample, on the other hand, is the highest value measured among the investigated samples; thus, this proxy does not represent a non-marine signal, whereas Ca concentration in the Italian rock salt sample also shows the same low value as the Kalahari salt, most probably because this salt has been refined. The significant differences in element concentrations (high U, absence of Ca, and very low concentrations of Mg and K) strongly support the interpretation of the Kalahari/South Africa salt sample to be of non-marine origin. Still, one has to keep in mind that sea salt precipitation occurs at the transition between sea and continent. Therefore, marine rock salt might also possess dominantly continental geochemical patterns if there was a strong influence/contamination with continental sediment during the period of formation. However, the present sea salt samples demonstrate that Ca, K, and Mg always co-precipitate with the salt; thus, it can be regarded as a good indicator for a marine genesis (also see [23]), and the low concentrations of these elements in the Kalahari salt sample as strong support of the assumption of its non-marine genesis.

The Dead Sea salt sample is extremely enriched with respect to the other salt samples in the following elements: K, Mg, As (the only sample over the detection limit), Se, and Rb, and also possesses the highest concentration of all samples analyzed for Cs and Mo. This clearly evidences the non-marine origin and the possibility to discriminate it from the other samples. However, U was not detected in the Dead Sea salt. Thus, it seems that the elevated U concentration in the Kalahari salt sample result is due to the geology in Southern Africa, and is not a general feature of non-marine salts. Logically, a non-marine salt, which exclusively originates from a catchment area consisting of a bedrock geology of only marine sediments (e.g., carbonates) should exhibit a “marine pattern” of element concentrations and, thus, no elevated U content, but this needs to be checked and confirmed.

**Figure 6:**
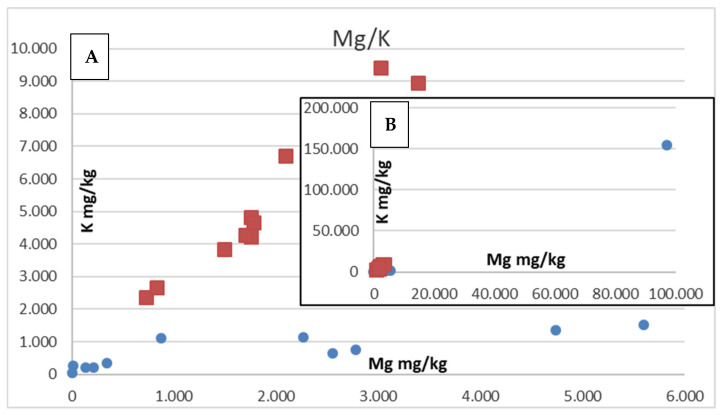
Squares identify the Austrian rock salt samples, circles show all other salt samples. (**A**) The Austrian salt samples show a differing trend in Mg/K concentrations with respect to the other salt samples. (**B**) The circle in the figure inlay shows the highly elevated values of the Dead Sea salt sample with respect to all other samples. (**A**) only shows a very small part of the inlay (**B**).

There are some distinct differences between the sea salt and the rock salt samples, and also between the four rock salt samples. Concerning rock salt, these differences most likely originate from the fact that the Toskana/Tuscany rock/table salt has been refined and thus many contaminants were removed (Ca, Mg, potentially Mn, Sr), and in this way can be identified within the group of investigated salt samples. The Himalaya salt (Pakistan) can be differentiated from the two Austrian rock salt samples due to lower Ba, Mg, K, Ca, Rb, and Sr values. The Ba and Ca concentrations in the Austrian rock salt samples are the highest of all the samples analyzed, and the K concentrations are second to the Dead Sea salt sample. This suggests that the deposition of this salt did not occur by evaporation of “normal” sea water but in a restricted environment allowing the concentration of certain elements. Correlation of Mg and K concentrations (Figure 6) shows a deviating trend of the Austrian salt samples with respect to the other salt samples, except for the Dead Sea salt sample that shows a similar Mg/K ratio, but with much higher values.

**Figure 7:**
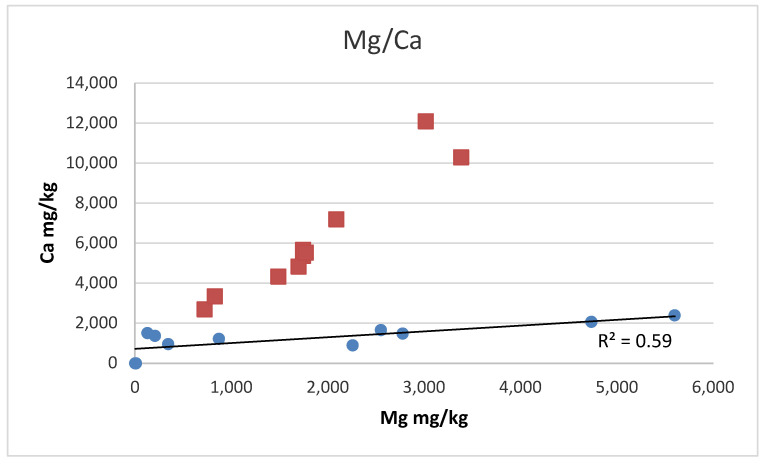
Mg to Ca concentrations in the salt samples. Squares identify the Austrian rock salt samples, circles show all other salt samples. The Austrian salt samples show a differing trend in Mg/Ca concentrations, with respect to the other salt samples. The Dead Sea salt sample is not shown in the figure, as it contains 97,200 mg/kg Mg and 568 mg/kg Ca.

In addition, also for the correlation of Ca and Mg, the Austrian samples show a deviating trend with respect to the other salt samples (Figure 7). The Dead Sea sample, however, deviates from the Austrian salt sample trend and the trend of the other salt samples. Thus, differences in the respective genesis can also be assumed for the Austrian and the Dead Sea salt samples.

The sea salt samples can partially be differentiated from each other by the following elements: the Korean sea salt sample by its measurable Cd and elevated Mn content; the Portuguese (Algarve) sample by the highest B value and the highest Mg, K, and Ca contents among the sea salt samples; the Spanish (Ibiza) salt sample by its lowest concentrations in Li, Mg, and Mn, among the sea salt samples; the New Zealand salt sample by the highest Al, Ni, and Sr concentrations in sea salt; and the sea salt sample from Sardinia/Italy by the highest Pb value.

It is interesting to note that the Mediterranean Sea salt samples have lower Rb, Li and Mo values than the other sea salt samples.

## 5. Conclusions

The sea salt samples can be differentiated mainly by variations in trace element concentrations. However, as single individual samples have been analyzed, the range of variation within one salt production plant will be important to assess which elemental variations are beyond a (possibly slight) heterogeneity in each production plant. The pattern in sulfur isotopes with slightly higher values for the Mediterranean, with respect to the other sea salt samples, is interesting but needs to be investigated further. A potential explanation might be the precipitation of sulfides in oxygen-deficient water depths, perhaps documenting the influence of Black Sea water, as the Black Sea is known to have an enlarged oxygen minimum zone. The rock salt samples have different sulfur isotope values due to different ages of salt precipitation; thus, mirroring the sulfur isotope composition of the respective sea water, which changed in Earth’s evolution. One non-marine salt sample from southern Africa can easily be distinguished from the other salt samples by its peculiar trace element pattern, but not by its δ^34^S value, which is within the marine δ^34^S range. Confirmation of the element concentration patterns found in the individual samples is needed by analysis of further salt samples to verify that the observed patterns are reproducible. The δ^34^S value of salt is a powerful proxy to identify sea salt samples, or to discriminate them from rock salt samples.

## Figures and Tables

**Table 1:** List and description of investigated salt samples.

Sample No.	Sample Type and Origin
159697	Rock salt, Kalahari salt, South Africa
159698	Sea salt, Mauritius
159699	Sea salt Sardinia, Italy
159700	Sea salt, Savoia, Italy
159701	Table salt (refined), Tuscany, Italy
159702	Rock salt, Himalaya salt, Pakistan
159703	Sea salt, Korea
159704	Sea salt, New Zeeland
159705	Sea salt, Algarve, Portugal
159706	Sea salt, Ibiza, Spain
155810-1	Rock salt, Rotes Kernsalz, Rotsalzgebirge, Altaussee, “AA”-subsample 1, Austria
155810-2	Rock salt, Rotes Kernsalz, Rotsalzgebirge, Altaussee, “AA”-subsample 2 Austria
155810-1-re-precipitated AA1	
155810-2-re-precipitated AA2	
155810 “AA“-subsample 3 milled in a corund mill, Austria	
155811 “HA“-subsample 3 milled in a corund mill, Austria	
155811-1	Rock salt, Rötlichgraues Kernsalz, Rotsalzgebirge, Hallein, “HT”-subsample 1, Austria
155811-2	Rock salt, Rötlichgraues Kernsalz, Rotsalzgebirge, Hallein, “HT”-subsample 2, Austria
155811-1-re-precipitated HT1	
155811-2-re-precipitated HT2	
155070	Sea salt, Slovenia
155599	Dead Sea salt, Israel

## Data Availability

All data are presented in the article.

## Supplementary Materials

The following supporting information can be downloaded at: https://www.mdpi.com/article/10.3390/foods12081572/s1, Table S1: Results of δ34S and element concentrations of the salt samples investigated in this study.
