# Supplementary material for: δ34S and Geochemical Analyses for the Determination of, and Discrimination between, Salt Samples of Different Geographic Origin: A Feasibility Study
Source: Foods. 2023 Apr 7;12(8):1572. doi: 10.3390/foods12081572 (PMC10138183; doi:10.3390/foods12081572)
Supplement: Supplementary file 1 [file foods-12-01572-s001.zip › foods-2287002-supplementary.pdf]

Externe ProbenNr:		159697	159698	159699	159700	159701	159702	159703	159704
		Kalaharisalz(S-A	Meersalz (Mauri	Meersalz Sardin	Meersalz, Savoi	Tafelsalz, Toska	Steinsalz Himala	Meersalz, Korea	Meersalz, Neuse
		Kalahari/South A	Mauritius	Sardinia/Italy	Savoia/Italy	Tuscany/Italy	Himalaya/Pakist	Korea	New Zealand
		rocksalt cont.	seasalt	seasalt	seasalt	rocksalt	rocksalt	seasalt	seasalt
Interne ProbenNr:		UF - 945 / 2009	UF - 946 / 2009	UF - 947 / 2009	UF - 948 / 2009	UF - 949 / 2009	UF - 950 / 2009	UF - 951 / 2009	UF - 952 / 2009
d34S	permil	18.7	20.3	21.8	22.0	17.0	27.7	21.1	21.1
Li	mg/kg	<0,05	0.43	0.06	0.09	0.21	0.29	0.56	0.38
Be	mg/kg	<0,1	<0,1	<0,1	<0,1	<0,1	<0,1	<0,1	<0,1
B	mg/kg	<10	<10	<10	<10	<10	<10	12	<10
Mg	mg/kg	4	2260	208	344	11	873	4737	2777
Al	mg/kg	<2	<2	<2	<2	<2	12	10	19
K	mg/kg	56	1147	214	353	275	1113	1360	760
Ca	mg/kg	<50	906	1380	963	<50	1233	2077	1497
Cr	mg/kg	<2	<2	<2	<2	<2	<2	<2	<2
Fe	mg/kg	<50	<50	<50	<50	<50	<50	<50	<50
Mn	mg/kg	<0,2	1.5	1.9	1.4	<0,2	0.5	4.2	1.4
Co	mg/kg	<0,02	0.02	0.02	<0,02	<0,02	0.03	0.02	0.03
Ni	mg/kg	<0,5	<0,5	<0,5	<0,5	<0,5	<0,5	0.6	0.7
Cu	mg/kg	<0,5	<0,5	<0,5	<0,5	<0,5	<0,5	<0,5	<0,5
Zn	mg/kg	<5	<5	<5	<5	<5	<5	<5	<5
Ga	mg/kg	<0,1	<0,1	<0,1	<0,1	<0,1	<0,1	<0,1	<0,1
As	mg/kg	<1	<1	<1	<1	<1	<1	<1	<1
Se	mg/kg	<5	<5	<5	<5	<5	<5	<5	<5
Rb	mg/kg	<0,05	0.34	<0,05	<0,05	<0,05	0.06	0.29	0.19
Sr	mg/kg	0.4	37	64	48	0.1	15	74	107
Y	mg/kg	<0,01	<0,01	<0,01	<0,01	<0,01	<0,01	<0,01	<0,01
Nb	mg/kg	<0,01	<0,01	<0,01	<0,01	<0,01	<0,01	<0,01	<0,01
Mo	mg/kg	0.12	0.04	<0,02	<0,02	<0,02	<0,02	0.05	0.09
Ag	mg/kg	<0,05	<0,05	<0,05	<0,05	<0,05	<0,05	<0,05	<0,05
Cd	mg/kg	<0,02	<0,02	<0,02	<0,02	<0,02	<0,02	0.02	<0,02
Sn	mg/kg	<0,03	<0,03	<0,03	<0,03	<0,03	<0,03	<0,03	<0,03
Sb	mg/kg	<0,01	<0,01	<0,01	<0,01	<0,01	<0,01	<0,01	<0,01
Te	mg/kg	<0,05	<0,05	<0,05	<0,05	<0,05	<0,05	<0,05	<0,05
Cs	mg/kg	<0,01	<0,01	<0,01	<0,01	<0,01	<0,01	<0,01	<0,01
Ba	mg/kg	<0,1	0.2	0.5	0.2	0.1	0.2	0.4	0.4
La	mg/kg	<0,01	<0,01	<0,01	<0,01	<0,01	<0,01	<0,01	0.02
Ce	mg/kg	<0,01	<0,01	<0,01	<0,01	<0,01	0.02	0.02	0.04
Pr	mg/kg	<0,01	<0,01	<0,01	<0,01	<0,01	<0,01	<0,01	<0,01
Nd	mg/kg	<0,05	<0,05	<0,05	<0,05	<0,05	<0,05	<0,05	<0,05
Sm	mg/kg	<0,01	<0,01	<0,01	<0,01	<0,01	<0,01	<0,01	<0,01
Eu	mg/kg	<0,01	<0,01	<0,01	<0,01	<0,01	<0,01	<0,01	<0,01
Gd	mg/kg	<0,01	<0,01	<0,01	<0,01	<0,01	<0,01	<0,01	<0,01
Tb	mg/kg	<0,01	<0,01	<0,01	<0,01	<0,01	<0,01	<0,01	<0,01
Dy	mg/kg	<0,01	<0,01	<0,01	<0,01	<0,01	<0,01	<0,01	<0,01
Ho	mg/kg	<0,01	<0,01	<0,01	<0,01	<0,01	<0,01	<0,01	<0,01
Er	mg/kg	<0,01	<0,01	<0,01	<0,01	<0,01	<0,01	<0,01	<0,01
Tm	mg/kg	<0,01	<0,01	<0,01	<0,01	<0,01	<0,01	<0,01	<0,01
Yb	mg/kg	<0,01	<0,01	<0,01	<0,01	<0,01	<0,01	<0,01	<0,01
Lu	mg/kg	<0,01	<0,01	<0,01	<0,01	<0,01	<0,01	<0,01	<0,01
W	mg/kg	<0,1	<0,1	<0,1	<0,1	<0,1	<0,1	<0,1	<0,1
Tl	mg/kg	<0,01	<0,01	<0,01	<0,01	<0,01	<0,01	<0,01	<0,01
Pb	mg/kg	0.1	0.2	0.6	<0,1	<0,1	<0,1	0.2	<0,1
Bi	mg/kg	<0,5	<0,5	<0,5	<0,5	<0,5	<0,5	<0,5	<0,5
U	mg/kg	0.10	<0,01	<0,01	<0,01	<0,01	<0,01	0.01	<0,01

**159705**	**159706**	**155810-1**	**155810-2**	**155810-1-einged**	**155810-2-einged**	**155810 Korundm**	**155811 Korundm**	**155811-1**	**155811-2**
**Meersalz, Algarv**	**Meersalz, Ibiza,**	**Altaussee**	**Altaussee**	**precipitated**	**precipitated**	**corund mill**	**corund mill**	**Hallein**	**Hallein**
**Algarve/Portuga**	**Ibiza/Spain**	**Austria AA-1**	**Austria AA-2**	**Austria AA-1**	**Austria AA-2**	**Austria AA-3**	**Austria HA-3**	**Austria HA-1**	**Austria HA-2**
**seasalt**	**seasalt**	**rocksalt**	**rocksalt**	**rocksalt-precip.**	**rocksalt-precip.**	**rocksalt-corund**	**rocksalt-corund**	**rocksalt**	**rocksalt**
**UF - 953 / 2009**	**UF - 954 / 2009**	**UF - 36 / 2010**	**UF - 37 / 2010**	**UF - 38 / 2010**	**UF - 39 / 2010**	**UF - 40 / 2010**	**UF - 41 / 2010**	**UF - 42 / 2010**	**UF - 43 / 2010**
20.7	21.7	11.7	12.2					11.3	11.3
0.66	<0,05	0.15	<0,05	0.10	<0,05	0.30	0.18	0.16	0.21
<0,1	<0,1	<0,1	<0,1	<0,1	<0,1	<0,1	<0,1	<0,1	<0,1
18	<10	<10	<10	<10	<10	<10	<10	<10	<10
5600	129	2090	725	3020	830	3383	1747	1700	1490
3	<2	46	11	<2	<2	71	60	27	36
1527	206	6717	2377	9420	2663	8957	4217	4267	3850
2403	1513	7200	2700	12100	3350	10300	5373	4837	4333
<2	<2	<2	<2	<2	<2	<2	<2	<2	<2
<50	<50	94	65	<50	<50	138	<50	<50	<50
1.4	<0,2	4.4	2.0	1.9	0.9	5.7	0.9	0.6	0.7
0.02	<0,02	0.06	0.03	0.04	0.03	0.09	0.04	0.03	0.03
<0,5	<0,5	<0,5	<0,5	<0,5	<0,5	<0,5	<0,5	<0,5	<0,5
<0,5	<0,5	<0,5	<0,5	<0,5	<0,5	0.7	<0,5	<0,5	<0,5
<5	<5	<5	<5	<5	<5	<5	<5	<5	<5
<0,1	<0,1	<0,1	<0,1	<0,1	<0,1	<0,1	<0,1	<0,1	<0,1
<1	<1	<1	<1	<1	<1	<1	<1	<1	<1
<5	<5	<5	<5	<5	<5	<5	<5	<5	<5
0.39	<0,05	1.2	0.4	1.1	0.4	1.2	0.2	0.2	0.2
80	52	110	78	156	91	158	141	144	125
<0,01	<0,01	0.04	0.01	<0,01	<0,01	0.05	0.03	0.01	0.02
<0,01	<0,01	<0,01	<0,01	<0,01	<0,01	<0,01	<0,01	<0,01	<0,01
0.06	<0,02	0.08	0.05	<0,02	<0,02	0.13	0.03	0.03	0.03
<0,05	<0,05	<0,05	<0,05	<0,05	<0,05	<0,05	<0,05	<0,05	<0,05
<0,02	<0,02	<0,02	<0,02	<0,02	<0,02	<0,02	<0,02	<0,02	<0,02
<0,03	<0,03	<0,03	<0,03	<0,03	<0,03	<0,03	<0,03	<0,03	<0,03
<0,01	<0,01	0.03	0.01	0.01	<0,01	0.04	<0,01	<0,01	<0,01
<0,05	<0,05	<0,05	<0,05	<0,05	<0,05	<0,05	<0,05	<0,05	<0,05
<0,01	<0,01	0.11	<0,01	0.06	<0,01	0.05	<0,01	<0,01	<0,01
0.1	<0,1	4.0	1.1	3.2	2.1	3.9	2.8	2.4	2.4
<0,01	<0,01	0.02	<0,01	0.01	<0,01	0.02	0.02	0.02	0.01
<0,01	<0,01	0.06	0.02	0.03	0.01	0.05	0.05	0.07	0.05
<0,01	<0,01	<0,01	<0,01	<0,01	<0,01	<0,01	<0,01	<0,01	<0,01
<0,05	<0,05	<0,05	<0,05	<0,05	<0,05	<0,05	<0,05	<0,05	<0,05
<0,01	<0,01	0.01	<0,01	<0,01	<0,01	0.01	<0,01	<0,01	<0,01
<0,01	<0,01	<0,01	<0,01	<0,01	<0,01	<0,01	<0,01	<0,01	<0,01
<0,01	<0,01	0.01	<0,01	<0,01	<0,01	0.02	<0,01	<0,01	<0,01
<0,01	<0,01	<0,01	<0,01	<0,01	<0,01	<0,01	<0,01	<0,01	<0,01
<0,01	<0,01	<0,01	<0,01	<0,01	<0,01	<0,01	<0,01	<0,01	<0,01
<0,01	<0,01	<0,01	<0,01	<0,01	<0,01	<0,01	<0,01	<0,01	<0,01
<0,01	<0,01	<0,01	<0,01	<0,01	<0,01	<0,01	<0,01	<0,01	<0,01
<0,01	<0,01	<0,01	<0,01	<0,01	<0,01	<0,01	<0,01	<0,01	<0,01
<0,01	<0,01	<0,01	<0,01	<0,01	<0,01	<0,01	<0,01	<0,01	<0,01
<0,01	<0,01	<0,01	<0,01	<0,01	<0,01	<0,01	<0,01	<0,01	<0,01
<0,1	<0,1	<0,1	<0,1	<0,1	<0,1	<0,1	<0,1	<0,1	<0,1
<0,01	<0,01	0.04	0.03	0.03	0.03	0.04	<0,01	<0,01	<0,01
0.2	0.1	0.5	0.1	0.2	<0,1	0.5	0.2	0.2	0.1
<0,5	<0,5	<0,5	<0,5	<0,5	<0,5	<0,5	<0,5	<0,5	<0,5
<0,01	<0,01	0.03	<0,01	<0,01	<0,01	0.03	0.02	0.02	0.02

155811-1-einged	155811-2-einged	155070	155599		
precipitated	preciptated	eersalz Slowenie	Totes Meer Salz		
Austria HA-1	Austria HA-2	Slovenia	Dead Sea Israel		
rocksalt-precip.	rocksalt-precip	seasalt	salt cont.		
UF - 44 / 2010	UF - 45 / 2010	UF - 46 / 2010	UF - 47 / 2010	Minimum	Maximum
				(above LOQ)	
		22.1	13.0		
0.10	0.09	0.31	0.37	**0.06**	**0.66**
<0,1	<0,1	<0,1	<0,1	**0.00**	**0.00**
<10	<10	<10	<10	**12.10**	**18.47**
1747	1773	2553	97200	**3.97**	**97200.00**
<2	<2	3	7	**3.17**	**71.07**
4827	4650	648	154667	**56.47**	**154666.67**
5680	5540	1667	568	**567.67**	**12100.00**
<2	<2	<2	<2	**0.00**	**0.00**
<50	<50	<50	<50	**64.90**	**137.67**
0.2	0.3	1.7	2.3	**0.24**	**5.69**
0.03	0.03	<0,02	0.03	**0.02**	**0.09**
<0,5	<0,5	0.32	0.16	**0.16**	**0.73**
<0,5	<0,5	<0,5	<0,5	**0.67**	**0.67**
<5	<5	<5	<5	**0.00**	**0.00**
<0,1	<0,1	<0,1	<0,1	**0.00**	**0.00**
<1	<1	<1	7.74	**7.74**	**7.74**
<5	<5	1.61	28.4	**1.61**	**28.40**
0.2	0.1	0.21	139.00	**0.06**	**139.00**
153	164	62	8	**0.11**	**164.00**
<0,01	<0,01	<0,01	<0,01	**0.01**	**0.05**
<0,01	<0,01	<0,01	<0,01	**0.00**	**0.00**
<0,02	<0,02	0.02	0.19	**0.02**	**0.19**
<0,05	<0,05	<0,05	<0,05	**0.00**	**0.00**
<0,02	<0,02	<0,02	<0,02	**0.02**	**0.02**
<0,03	<0,03	<0,03	<0,03	**0.00**	**0.00**
<0,01	<0,01	<0,01	<0,01	**0.01**	**0.04**
<0,05	<0,05	<0,05	<0,05	**0.00**	**0.00**
<0,01	<0,01	<0,1	0.38	**0.05**	**0.38**
3.7	3.6	0.7	0.15	**0.12**	**4.04**
<0,01	0.02	<0,1	<0,1	**0.01**	**0.02**
0.02	0.05	<0,1	<0,1	**0.01**	**0.07**
<0,01	<0,01	<0,01	<0,01	**0.00**	**0.00**
<0,05	<0,05	<0,05	<0,05	**0.00**	**0.00**
<0,01	<0,01	<0,01	<0,01	**0.01**	**0.01**
<0,01	<0,01	<0,01	<0,01	**0.00**	**0.00**
<0,01	<0,01	<0,01	<0,01	**0.01**	**0.02**
<0,01	<0,01	<0,01	<0,01	**0.00**	**0.00**
<0,01	<0,01	<0,01	<0,01	**0.00**	**0.00**
<0,01	<0,01	<0,01	<0,01	**0.00**	**0.00**
<0,01	<0,01	<0,01	<0,01	**0.00**	**0.00**
<0,01	<0,01	<0,01	<0,01	**0.00**	**0.00**
<0,01	<0,01	<0,01	<0,01	**0.00**	**0.00**
<0,01	<0,01	<0,01	<0,01	**0.00**	**0.00**
<0,1	<0,1	<0,1	<0,1	**0.00**	**0.00**
<0,01	<0,01	<0,01	<0,01	**0.03**	**0.04**
<0,1	1.3	<0,1	<0,1	**0.12**	**1.28**
<0,5	<0,5	<0,5	<0,5	**0.00**	**0.00**
<0,01	<0,01	<0,01	<0,01	**0.01**	**0.10**
